# Interactions between meat intake and genetic variation in relation to colorectal cancer

**DOI:** 10.1007/s12263-014-0448-9

**Published:** 2014-12-10

**Authors:** Vibeke Andersen, Ulla Vogel

**Affiliations:** 1 Organ Center, Hospital of Southern Jutland, Aabenraa, Denmark; 2 Institute of Regional Health Research, University of Southern Denmark, Odense, Denmark; 3 Medical Department, Regional Hospital Viborg, Viborg, Denmark; 4 National Research Centre for the Working Environment, Copenhagen, Denmark

**Keywords:** Colorectal carcinogenesis, Genetic susceptibility, Genetic epidemiology, Polymorphisms, Gene–environment interactions, Diet–gene interactions, Lifestyle

## Abstract

Meat intake is associated with the risk of colorectal cancer. The objective of this systematic review was to evaluate interactions between meat intake and genetic variation in order to identify biological pathways involved in meat carcinogenesis. We performed a literature search of PubMed and Embase using “interaction”, “meat”, “polymorphisms”, and “colorectal cancer”, and data on meat–gene interactions were extracted. The studies were divided according to whether information on meat intake was collected prospectively or retrospectively. In prospective studies, interactions between meat intake and polymorphisms in *PTGS2* (encoding COX-2), *ABCB1*, *IL10*, *NFKB1*, *MSH3*, *XPC* (*P*_int_ = 0.006, 0.01, 0.04, 0.03, 0.002, 0.01, respectively), but not *IL1B*, *HMOX1*, *ABCC2*, *ABCG2*, *NR1I2* (encoding PXR), *NR1H2* (encoding LXR), *NAT1*, *NAT2*, *MSH6*, or *MLH1* in relation to CRC were found. Interaction between a polymorphism in *XPC* and meat was found in one prospective and one case–control study; however, the directions of the risk estimates were opposite. Thus, none of the findings were replicated. The results from this systematic review suggest that genetic variation in the inflammatory response and DNA repair pathway is involved in meat-related colorectal carcinogenesis, whereas no support for the involvement of heme and iron from meat or cooking mutagens was found. Further studies assessing interactions between meat intake and genetic variation in relation to CRC in large well-characterised prospective cohorts with relevant meat exposure are warranted.

**Electronic supplementary material:**

The online version of this article (doi:10.1007/s12263-014-0448-9) contains supplementary material, which is available to authorized users.

## Introduction

Colorectal cancer (CRC) is a major health problem worldwide. In the Western World, CRC is the third most common cancer and the one with the second highest mortality (WCRF 2014). In the developing countries, the incidence is increasing due to demographic changes and due to implementation of Western lifestyle. Lifestyle factors, including diet, are considered to be the main causes of CRC (WCRF 2014). High intake of red and processed meat, animal fat, alcohol, and smoking is the factor that has been associated with the risk of CRC, whereas high intake of dietary fibres, fruit and vegetables, and physical activity is considered to protect from CRC (Huxley et al. 2009; WCRF 2014). The World Cancer Research Fund has evaluated observational and experimental evidence linking the intake of red and processed meat to CRC as convincing (WCRF 2014). Furthermore, they judged that half of all CRC cases may be prevented by relevant lifestyle changes (WCRF 2014). Accordingly, advancing the understanding of underlying mechanisms for developing CRC may have large implications for human health by forming the basis for preventive interventions.

**Fig. 1:**
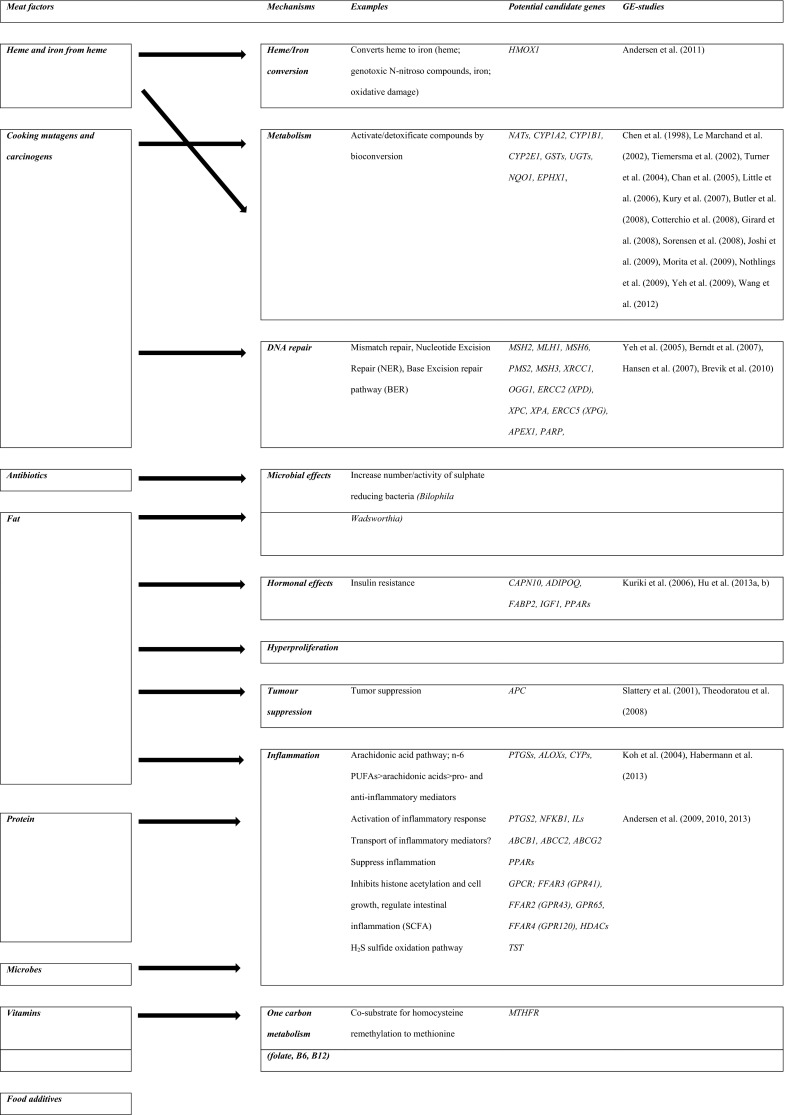
Examples of potential mechanisms by which meat may affect colorectal carcinogenesis

Various mechanisms by which intake of red and processed meat may promote colorectal carcinogenesis have been suggested (Santarelli et al. 2008; Ferguson 2010; Alexander and Cushing 2011; Alexander et al. 2011; Chan et al. 2011; Erridge 2011; Zur 2012). Meat is a source of fat, protein, dietary iron, zinc, sulphur, and vitamins and may contain microbes developed during storage, various additives, cooking mutagens, and antibiotics. These meat compounds may be carcinogenic by various mechanisms as illustrated in Fig. 1. For example, heterocyclic amines (HAC), polycyclic aromatic hydrocarbons (PAH), and *N*-nitroso compounds (NOC) present in meat or arising during processing and cooking at high temperature may introduce DNA damage leading to the generation of mutations and cancer (Santarelli et al. 2008). The carcinogenic effects will depend on the efficiency of the human metabolism of the compound (activation, degradation, or excretion) and on the efficiency of repair of the DNA damage (Fig. 1). Hence, HCAs may be activated by *N*-acetyltransferases (encoded by *NAT1* and *NAT2*) to form carcinogens acting in the colon epithelium, whereas phase II xenobiotic metabolising enzymes such as UDP-glucuronosyltransferases (encoded by the UGTs) may detoxify the cooking carcinogens (Gilsing et al. 2012; Ollberding et al. 2012). Also, protein fermentation by the colonic bacteria may lead to the formation of carcinogenic substances such as hydrogen sulphide (H_2_S) (Hamer et al. 2012; Windey et al. 2012; Andersen 2014a). In particular, meat contains high amounts of fat and proteins, including organic sulphur-containing proteins, which may contribute to enhance the microbial production of H_2_S. This leads to DNA damage, up-regulation of pro-inflammatory COX-2, and suppression of anti-inflammatory butyrate. Thus, a diet high in animal fat was found to increase the amount and activity of the *Bilophila Wadsworthia* in an animal model (Devkota et al. 2012). Because this bacterium reduces sulphite (SO_3_^2−^) from diet to H_2_S by anaerobic oxidation and because meat is a particularly rich source of organic sulphur, this results in high colonic production of H_2_S (Carbonero et al. 2012). Besides inducing DNA damage, H_2_S and its ion sulphide (S^2−^) has been associated with the up-regulation of COX-2; impaired oxidation of butyrate, which is the most important fuel in the intestinal cells (Windey et al. 2012); and induction of intestinal hyperproliferation (Carbonero et al. 2012). Thus, meat intake, intestinal microbes, and individual factors may interact and affect intestinal inflammation (Jia et al. 2012). Furthermore, a diet high in fat may increase the risk of CRC by hormonal mechanisms (Fig. 1). Moreover, n-6 polyunsaturated fatty acids (n-6 PUFAs) from meat are converted into arachidonic acid that is further metabolised by the cytochrome P450 oxygenase (CYP), the cyclooxygenase (COX), and the lipoxygenase (LOX) pathways to pro- and anti-inflammatory prostaglandins (PG) and leukotrienes (LT) including PGE_2_ and LTB_4_, which have been found to be involved in colorectal carcinogenesis (Wang and DuBois 2010a, b; 2013). Also, indications that microbial factors present in meat or arising during storage may be involved in CRC have been found in (Erridge 2011; Zur 2012). Thus, intake of meat may potentially affect intestinal homeostasis by a range of various mechanisms leading to somatic mutations, epigenetic changes, and impaired balance between proliferation and apoptosis resulting in cancer development as summarised in Fig. 1.

Genetically determined variations in the activity of enzymes or pathways may modify the processes mentioned in Fig. 1 and thereby influence meat-related risk of CRC. Hence, assessment of gene–environment interactions provides a tool to identify the combinations of genes and environmental factors involved in CRC because the presence of an interaction indicates that the two factors are involved in the same process (Vogel et al. 2007; Andersen et al. 2009, 2010, 2012a, b, 2013a, b). Furthermore, use of functional polymorphisms, i.e. polymorphisms which lead to changed protein activity, may help the biological understanding. Gene–environment interaction studies may generate knowledge on biological mechanisms and may provide indications for primary prevention. In gene–environment interaction studies, human metabolism and the complexity of lifestyle factors are taken into account. This is difficult to achieve by other means. We therefore reviewed the literature on interactions between meat intake and polymorphisms in relation to CRC in order to identify pathways involved in the effects of meat intake.

## Methods

**Fig. 2:**
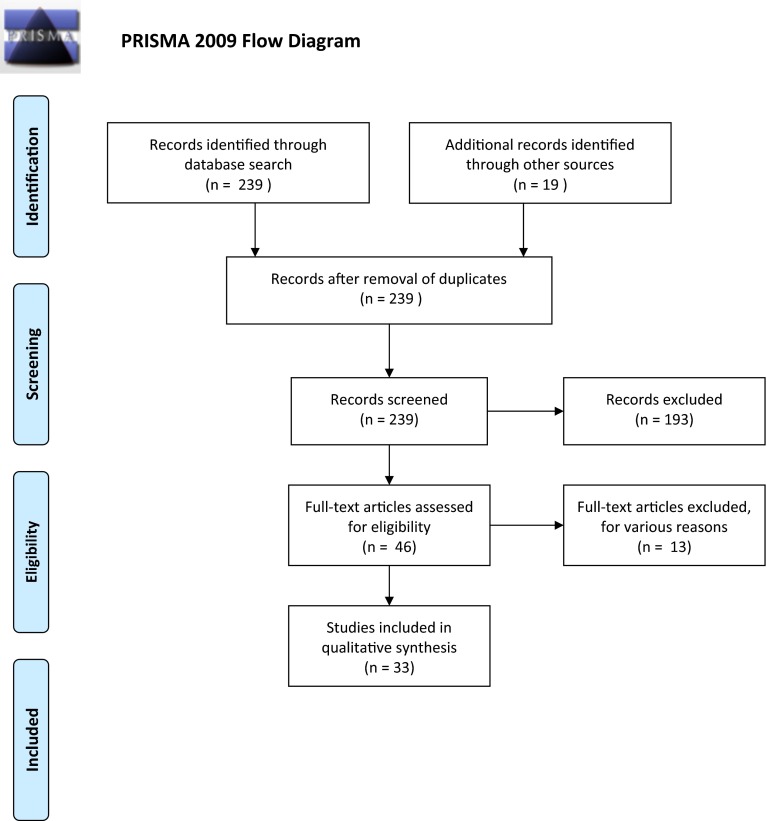
Preferred reporting items for systematic reviews and meta-analyses (PRISMA) flow diagram of the retrieved studies

A systematic review was carried out according to the guidelines of Preferred Reporting Items for Systematic Reviews and Meta-Analyses (PRISMA) statement (Moher et al. 2009) (Fig. 2). PubMed and Embase were searched for various combinations of “meat”, “colorectal cancer”, “snp(s)”, “gene variant”, and “polymorphisms” [e.g. (“red and processed meat” OR “red meat” OR “processed meat” OR “meat”) AND “colorectal cancer” AND (“genetic” OR “polymorphism” OR “polymorphisms” OR “gene variants” OR “snps” OR “snp”)] with no restrictions (e.g. on years considered) resulting in 239 abstracts *in total* (January, May, and August 2014). Articles from abstracts suggesting that they presented original data on polymorphisms and meat interaction were retrieved and read. All studies which reported original data on meat intake and gene interactions and which were published in English were included.

Studies were excluded due to missing data on the interaction analyses between meat intake and gene variants in relation to CRC, interaction with meat-related variables (proxies), and not meat itself was performed and with less than 25 cases in the subgroup analyses.

Information on study design, the number of participants, incidence rate ratios (IRR) and odds ratios (OR), *P* value for interaction (*P*_int_) from the interaction analyses between meat intake and polymorphisms in relation to CRC was retrieved from the studies when present. When rs number was not provided by the authors, the rs number was retrieved using PubMed Gene (http://www.ncbi.nlm.nih.gov/gene/324) by selecting SNP gene view and provided when the rs number could be unambiguously identified. Furthermore, polymorphisms which deviated from Hardy–Weinberg equilibrium were excluded (one polymorphism).

*P*_int_ indicates whether there was statistically significant interaction between the effects of meat intake and genotypes in relation to the risk of CRC.

The retrieved studies were divided according to the time when the information on meat intake was sampled into prospective studies (data collected before the diagnosis of CRC, Table 1) and case–control studies (data collected after the diagnosis of CRC, Table S1). *P* values adjusted for confounders and not corrected for multiple testing were chosen whenever possible (Table 1, Table S1). *P* value below 0.05 was considered statistically significant.

**Table 1:** Interactions between meat intake and polymorphisms in relation to the risk of colorectal cancer in prospective cohorts

Gene	rs-number^d^			*N*_cases_	*N*_sub-cohort_	IRR/OR (95 % CI)^a^	*P*_int_^b^	Comments^c^	First author	Year	References
*Cooking carcinogens and mutagens*											
*NAT1*			Slow	120	123			4	Chen	1998	Chen et al. (1998)
		NAT*10 allele	Rapid	92	98		0.19	4			Chen et al. (1998)
*NAT2*			Slow	131	125			4			Chen et al. (1998)
			Rapid	81	96		0.56	4			Chen et al. (1998)
*NAT2*			Slow	107	267			5	Chan	2005	Chan et al. (2005)
			Rapid	76	476		0.07	5			Chan et al. (2005)
*NAT1*			Slow			0.99 (0.94–1.04)		2, 3, 6	Sorensen	2008	Sorensen et al. (2008)
			Fast			0.98 (0.90–1.05)	>0.40	2, 3, 6			Sorensen et al. (2008)
*NAT2*			Slow			1.00 (0.95–1.06)		2, 3, 6			Sorensen et al. (2008)
			Fast			0.96 (0.90–1.03)	>0.40	2, 3, 6			Sorensen et al. (2008)
*NAT1*			No*10	362	527			7	Nothlings	2009	Nothlings et al. (2009)
			*10	482	818		0.77	7			Nothlings et al. (2009)
*NAT2*			Slow/med	750	1149			7			Nothlings et al. (2009)
			Rapid	242	344		0.44	7			Nothlings et al. (2009)
*NAT1*			No*10	362	527			8			Nothlings et al. (2009)
			*10	482	818		0.93	8			Nothlings et al. (2009)
*NAT2*			Slow/med	750	1,149			8			Nothlings et al. (2009)
			Rapid	242	344		0.13	8			Nothlings et al. (2009)
*AHR*	rs2066853			364	394		0.07	12	Gilsing	2012	Gilsing et al. (2012)
*UGT1A*	rs6714486			364	394		0.06	12			Gilsing et al. (2012)
	rs17868299			364	394		0.05	12			Gilsing et al. (2012)
*UGT1A*	rs2011404			364	394		0.08	12			Gilsing et al. (2012)
*CYP2E1*	rs915908			364	394		0.05	12			Gilsing et al. (2012)
*UGT1A*	rs6717546			364	394		*0.04*	12			Gilsing et al. (2012)
*UGT1A*	rs12466997			364	394		0.08	12			Gilsing et al. (2012)
*Arachidonic acid pathway*											
*PTGS2 (COX*-*2)*	rs689566	A-1195G	AA–AG	900	1,686	1.02 (0.98–1.05)		1, 2, 3	Andersen	2013	Andersen et al. (2013b)
			GG	47	61	1.06 (0.87–1.29)	0.54	1, 2, 3			Andersen et al. (2013b)
	rs20417	G-765C	GG	701	1,256	0.99 (0.95–1.03)		1, 2, 3			Andersen et al. (2013b)
			GC–CC	235	478	1.08 (1.01–1.15)	*0.006*	1, 2, 3			Andersen et al. (2013b)
	rs5275	T8473C	TT	430	720	1.04 (0.99–1.09)		1, 2, 3			Andersen et al. (2013b)
			TC–CC	501	1,018	1.01 (0.96–1.05)	0.29	1, 2, 3			Andersen et al. (2013b)
*Transport proteins*											
*ABCB1 (MDR1)*	rs1045642	3435	CC	73	118	1.08 (1.00–1.16)		1, 2, 3	Andersen	2009	Andersen et al. (2009)
			CT–TT	286	647	1.00 (0.95–1.06)	*0.02*	1, 2, 3			Andersen et al. (2009)
	rs3789243	Intron 3	GG	81	224	0.95 (0.89–1.02)		1, 2, 3			Andersen et al. (2009)
			GA–AA	278	541	1.03 (0.98–1.09)	*0.01*	1, 2, 3			Andersen et al. (2009)
*ABCG2 (BCRP)*	rs2231142	C421A	CC	296	592	1.02 (0.97–1.08)		1, 2, 3			Andersen et al. (2009)
			CA–AA	63	173	0.99 (0.91–1.08)	0.40	1, 2, 3			Andersen et al. (2009)
*ABCC2 (MRP2)*	rs717620	C-24T	CC	260	508	1.02 (0.97–1.07)		1, 2, 3	Andersen	2012	Andersen et al. (2012b)
			CT–TT	129	280	1.03 (0.95–1.12)	0.72	1, 2, 3			Andersen et al. (2012b)
	rs2273697	G1249A	GG	238	480	1.05 (0.99–1.11)		1, 2, 3			Andersen et al. (2012b)
			AG–AA	151	308	0.98 (0.91–1.05)	0.10	1, 2, 3			Andersen et al. (2012b)
	rs3740066	C3972T	CC	143	301	1.01 (0.96–1.08)		1, 2, 3			Andersen et al. (2012b)
			CT–TT	246	487	1.03 (0.97–1.10)	0.69	1, 2, 3			Andersen et al. (2012b)
*Cytokines*											
*IL10*	rs1800872	C-592A	CC	238	470	1.02 (0.97–1.07)		1, 2, 3, 9	Andersen	2012	Andersen et al. (2012b)
			AC–AA	140	305	1.02 (0.95–1.11)	0.92	1, 2, 3, 9			Andersen et al. (2012b)
	rs3024505		CC	268	553	1.02 (0.96–1.08)		1, 2, 3, 9			Andersen et al. (2012b)
			CT–TT	110	222	1.03 (0.96–1.10)	0.78	1, 2, 3, 9			Andersen et al. (2012b)
*IL10*	rs1800872	C-592A	CC	596	1072	1.02 (0.98–1.06)		1, 2, 3	Andersen	2013	Andersen et al. (2013b)
			AC–AA	353	676	1.00 (0.95–1.06)	0.46	1, 2, 3			Andersen et al. (2013b)
	rs3024505		CC	648	1,200	1.00 (0.96–1.04)		1, 2, 3			Andersen et al. (2013b)
			CT–TT	297	565	1.06 (1.00–1.11)	*0.04*	1, 2, 3			Andersen et al. (2013b)
*IL1B*	rs4848306	C-3737T	CC	336	560	1.01 (0.96–1.07)		1, 2, 3			Andersen et al. (2013b)
			CT–TT	605	1,186	1.02 (0.98–1.06)	0.65	1, 2, 3			Andersen et al. (2013b)
	rs1143623	G-1464C	GG	454	925	1.02 (0.97–1.06)		1, 2, 3			Andersen et al. (2013b)
			GC–CC	492	824	1.02 (0.97–1.07)	0.94	1, 2, 3			Andersen et al. (2013b)
	rs1143627	T-31C	TT	389	773	1.00 (0.96–1.05)		1, 2, 3			Andersen et al. (2013b)
			TC–CC	557	983	1.03 (0.98–1.07)	0.40	1, 2, 3			Andersen et al. (2013b)
*Transcription factors*											
*NFKB1*	rs28362491	−94 ins/del	II	122	307	0.96 (0.90–1.04)		1, 2, 3	Andersen	2010	Andersen et al. (2010)
			ID–DD	261	456	1.03 (0.97–1.08)	0.03	1, 2, 3			Andersen et al. (2010)
*NR1I2 (PXR)*	rs1523127	A-24381C	AA	131	261	1.04 (0.97–1.12)		1, 2, 3			Andersen et al. (2010)
			AC–CC	252	502	1.01 (0.95–1.06)	0.20	1, 2, 3			Andersen et al. (2010)
	rs2276707	C8055T	CC	237	448	1.02 (0.96–1.08)		1, 2, 3			Andersen et al. (2010)
			CT–TT	146	315	1.01 (0.95–1.08)	0.74	1, 2, 3			Andersen et al. (2010)
	rs6785049	A7635G	AA	137	264	1.01 (0.95–1.07)		1, 2, 3			Andersen et al. (2010)
			AG–GG	246	499	1.02 (0.96–1.08)	0.60	1, 2, 3			Andersen et al. (2010)
*NR1H2 (LXR)*	rs1405655		CC	40	76	1.01 (0.93–1.10)		1, 2, 3			Andersen et al. (2010)
			CT–TT	343	687	1.02 (0.96–1.07)	0.94	1, 2, 3			Andersen et al. (2010)
	rs2695121		TT	117	227	1.03 (0.96–1.11)		1, 2, 3			Andersen et al. (2010)
			CT–CC	266	536	1.01 (0.96–1.07)	0.43	1, 2, 3			Andersen et al. (2010)
*Heme oxygenase*											Andersen et al. (2011a)
*HMOX1 (HO*-*1)*	rs2071746	A-413T	AA	118	260	1.00 (0.93–1.08)		1, 2, 3	Andersen	2011	Andersen et al. (2011a, b)
			AT–TT	265	503	1.02 (0.97–1.08)	0.55	1, 2, 3			Andersen et al. (2011a, b)
*DNA repair*											
*MSH3*	rs184967	R940Q	RR	127				8, 10	Berndt	2007	Berndt et al. (2007)
			RQ–QQ	65			0.08	8, 10			Berndt et al. (2007)
*MSH3*	rs26279	T1036A	TT	85				8, 10			Berndt et al. (2007)
			TA–AA	102			*0.002*	8, 10			Berndt et al. (2007)
*MSH6*	rs1042821	G39E	GG	118				8, 10			Berndt et al. (2007)
			GE–EE	54			0.29	8, 10			Berndt et al. (2007)
*MLH1*	rs1799977	I219V	II	84				8, 10			Berndt et al. (2007)
			IV–VV	101			0.40	8, 10			Berndt et al. (2007)
*XPC*	Rs2228001^d^	Lys939Gln	AA	141	307	1.17 (0.71–1.92)		7, 11	Hansen	2007	Hansen et al. (2007)
			AC	204	392	1.11 (0.70–1.75)		7, 11			Hansen et al. (2007)
			CC	50	98	3.70 (1.70–8.04)	*0.01*	7, 11			Hansen et al. (2007)
*XPA*		A23G	GG	176	339	1.30 (0.78–2.17)		7, 11			Hansen et al. (2007)
			AG	187	359	1.41 (0.87–2.26)		7, 11			Hansen et al. (2007)
			AA	31	90	0.76 (0.34–1.66)	0.37	7, 11			Hansen et al. (2007)
*ERCC2 (XPD)*	Rs1799793^d^	Asp312Asn	GG	159	333	1.25 (0.69–2.26)		7, 11			Hansen et al. (2007)
			AG	191	354	1.25 (0.83–1.87)		7, 11			Hansen et al. (2007)
			AA	46	108	1.22 (0.61–2.45)	1.00	7, 11			Hansen et al. (2007)
*XPC*	Rs2228001^d^	Lys939Gln	AA	141	307	0.63 (0.23–1.69)		8, 11			Hansen et al. (2007)
			AC	204	392	0.94 (0.41–2.15)		8, 11			Hansen et al. (2007)
			CC	50	98	3.78 (0.64–22.29)	0.20	8, 11			Hansen et al. (2007)
*XPA*		A23G	GG	176	339	0.58 (0.23–1.48)		8, 11			Hansen et al. (2007)
			AG	187	359	1.87 (0.73–4.83)		8, 11			Hansen et al. (2007)
			AA	31	90	0.31 (0.06–1.64)	0.06	8, 11			Hansen et al. (2007)
*ERCC2 (XPD)*	Rs1799793^d^	Asp312Asn	GG	159	333	1.07 (0.38–3.03)		8, 11			Hansen et al. (2007)
			AG	191	354	0.75 (0.33–1.68)		8, 11			Hansen et al. (2007)
			AA	46	108	1.93 (0.43–8.63)	0.49	8, 11			Hansen et al. (2007)

^a^Odds ratio (OR) or incidence rate ratio (IRR)

^b^ *P* for interaction (*P*_int_) between meat and the genotypes in relation to CRC has been retrieved from the studies and may have been calculated in different ways

^c^Comments: (1) Incidence rate ratio (IRR) for colorectal cancer per intake of 25 g red or processed meat. (2) Adjusted for sex and age, smoking status, alcohol, HRT status (women only), BMI, NSAID use, and dietary fibre. (3) *P* value for interaction between the genotype and dietary intake for the fully adjusted risk estimate. (4) Tertile analyses of daily red meat intake (≤0.5, >0.5–1, <1). 5) Tertile analyses of intake of beef, pork, or lamb as a main dish (≤0.5, >0.5 servings per day). (6) IRR for colorectal cancer per intake of 25 g red meat. (7) Intake of red meat. (8) Intake of processed meat. (9) These data (Andersen et al. 2012a, b) are also included in (Andersen et al. 2013a, b). (10) Adjusted for age, race, and energy intake. (11) Adjusted for average smoking intensity, intake of alcohol, fruits/vegetable, fish/poultry, red meat, dietary fibres, BMI, and hormone replacement therapy. (12) Interactions between intake of benzo{a]pyrene (B[a]P), 2-amino-3,8-dimethylimidazol[4,5-f]quinoxaline (MeIQx), 2-amino-3,4,8-trimethylimidazol[4,5-f]quinoxaline (DiMeIQx), and combined nitrate and nitrite, respectively, and the polymorphisms

^d^rs number was not provided by the authors. Rs number has been identified and provided as described in the “Methods” section in cases where the rs number could be unambiguously identified

Replication of found results in an independent cohort is an important tool to identify gene–environment interactions in genetic epidemiology (Andersen and Vogel 2014a, b). In the present work, identification of gene–environment interactions was performed in the prospective studies (discovery cohorts). We regarded the finding as replicated if the results were reproduced in another prospective study or in a case–control study.

Data from (Chen et al. 1998; Tiemersma et al. 2002; Sorensen et al. 2008; Chan et al. 2011) have been presented in a previous review (Andersen et al. 2013a, b).

## Results

Table 1 and Table S1 show results on interactions between meat intake and polymorphisms in relation to CRC from prospective and case–control studies, respectively.

### Cooking carcinogens and mutagens

Prospective studies have evaluated the interaction between fast and slow acetylators and meat intake in relation to the risk of CRC (Table 1) (Chen et al. 1998; Chan et al. 2005; Sorensen et al. 2008; Nothlings et al. 2009; Gilsing et al. 2012). Whereas one small study found interaction between the number of servings per day and *NAT2* acetylator status (Chan et al. 2005), no association was found between the amount of total or processed meat intake or number of servings and *NAT1* or *NAT2* status in relation to the risk of CRC in three other studies (Chen et al. 1998; Sorensen et al. 2008; Nothlings et al. 2009).

### Arachidonic acid pathway

Interaction between meat intake and the *PTGS2* G-765C (rs20417) polymorphisms was found in a prospective study (*P*_int_ = 0.006) (Table 1) (Andersen et al. 2013a, b). Thus, individuals carrying the G-765C C-variant allele were at 8 % increased risk of CRC per 25 g red and processed meat per day in contrast to the homozygous wild-type carriers whose risk of CRC was unaffected by meat intake.

### Transport proteins

Interactions between meat intake and polymorphisms in *ABCB1* in relation to the risk of CRC were found in a prospective cohort, whereas no interactions were found for the two other transport proteins, *ABCC2* and *ABCG2* (Table 1) (Andersen et al. 2009, 2012a, b). Intake of meat was associated with increased risk among the *ABCB1* C3435T homozygous wild-type and intron 3 G-rs3789243-A-variant allele carriers, whereas the risk of CRC for carriers of the other alleles was unaffected by meat intake (Andersen et al. 2009).

### Cytokines

Interaction between meat intake and the marker polymorphism near *IL10* rs3024505 was found in a prospective cohort, whereas no interaction was found with the functional *IL10* C-592A nor with three functional *IL1B* polymorphisms (Andersen et al. 2013a, b) (Table 1).

### Transcription factors

No interactions were found between meat intake and the genes *NR1I2* and *NR1H2* encoding PXR and LXR in relation to CRC (Table 1) (Andersen et al. 2010). Interactions were found between meat intake and *NFKB1* (encoding the anti-inflammatory subunit p50/p105 of NFκB) −94 ins/del (rs2836249) in relation to the risk of CRC in a prospective cohort (Table 1) (Andersen et al. 2010). Carriers of the *NFKB1* −94ins/del del-variant alleles were at 3 % higher risk of CRC per 25 g meat eaten per day compared to homozygous wild-type allele carriers who had no risk by meat intake (Table 1).

### Heme oxygenase

No interactions were found between the functional *HMOX1* A-413T (rs2071746) polymorphism and meat intake in relation to CRC (Table 1) (Andersen et al. 2011a).

### DNA repair

A statistically significant interaction between the intake of processed meat and the mismatch repair gene *MSH3* T1036A (rs26279) and a suggestive interaction with R940Q (rs184967) was found in a prospective case-only study of approximately 185 persons (*P*_int_ = 0.002 and 0.08, respectively) (Table 1) (Berndt et al. 2007). Interpretation of the results was not possible because possible functional effects of the polymorphisms were not known (Berndt et al. 2007).

A statistically significant interaction between the intake of red meat and *XPC* Lys939Gln and a suggestive interaction between the intake of processed meat and *XPA* A23G was found in a prospective study (*P*_int_ = 0.01 and 0.06, respectively) (Hansen et al. 2007) (Table 1). Homozygous variant carriers of *XPC* Lys939Gln were at high risk of CRC by the intake of red meat compared to the homozygous wild-type carriers (reference) [IRR = 3.78 (1.70–8.04) and 1.17 (0.71–1.92] per 100 g of red meat per day, respectively, *P*_int_ = 0.01) (Hansen et al. 2007). The *XPC* Lys939Gln polymorphism was also identified in a case–control study (Steck et al. 2014) (Table S1). They found that homozygous wild-type carriers had an increased risk by high meat compared to low meat intake in the same group, whereas variant allele carriers had no increased risk by high meat intake [OR = 1.5 (1.0–2.2) and 1.0 (0.9–1.8] for homozygous wild-type carriers with high meat and low meat intake, respectively, *P*_int_ = 0.05) (Steck et al. 2014) (Table S1). Thus, in contrast to the study above, increased risk for high well-done red meat intake was found among homozygous wild-type carriers in the case–control study.

## Discussion

In this review, we evaluated gene–environment interactions between meat intake and genetic variation in relation to CRC in order to identify the biological pathways underlying meat-related CRC carcinogenesis (Fig. 1; Table 1, and S1). The retrieved studies were divided into prospective studies (Table 1) and case–control studies (Table S1) according to the risk of recall bias. We assessed whether found results were replicated in an independent cohort as this is considered an important tool to identify gene–environment interactions in genetic epidemiology.

The meat content of HCAs, PAHs, and NOCs has been suggested to confer the risk of CRC in humans (Santarelli et al. 2008; Ferguson 2010; Alexander and Cushing 2011; Alexander et al. 2011; Erridge 2011; Zur 2012). Prolonged high-temperature cooking of meat leads to the production of HCAs and PAHs, especially grilling, barbecuing, and frying (Ferguson 2010). In this review, we reported that one small study found interaction between the number of servings per day and *NAT2* acetylator status (Chan et al. 2005), whereas no association was found between the amount of total or processed meat intake or number of servings and *NAT1* or *NAT2* status in relation to the risk of CRC in three other studies (Chen et al. 1998; Sorensen et al. 2008; Nothlings et al. 2009). The results of this review are thus in accordance with a large prospective study of 1757 CRC cases found no association between the intake of HCA from meat and risk of CRC (Ollberding et al. 2012). Thus, gene–environment interaction studies do not support a strong role of HCAs in the aetiology of CRC.

*PTGS2* (encoding COX-2) is induced by inflammatory stimuli (Wang and DuBois 2010a, b). COX enzymes catalyse the rate-limiting conversion of arachidonic acid to prostaglandins such as the pro-inflammatory and pro-carcinogenic prostaglandin E_2_ (PGE_2_) (Wang and DuBois 2010a, b; Bacchi et al. 2012). In this review, we found that individuals carrying the G-765C C-variant allele were at high risk of CRC by the intake of meat in contrast to the homozygous wild-type carriers (Andersen et al. 2013a, b). The functional effect of the *PTGS2* G-765C polymorphisms is not clear as studies have found higher as well as lower activity associated with the variant (Papafili et al. 2002; Brosens et al. 2005; Zhang et al. 2005). In Danes, the *PTGS2* G-765C-variant allele is in tight linkage with the *PTGS2* T8473C-variant allele (Andersen et al. 2011b). The microRNA Mir-542-3p targets *PTGS2* mRNA for decay through binding to the T8473C wild-type allele, whereas the variant allele disrupts the binding leading to increased half-life of the *PTGS2* mRNA (Moore et al. 2012). This finding suggests that carriers of the variant alleles of these polymorphisms have a genetically determined high level of *PTGS2* mRNA. On the other hand, no interaction was found between the *PTGS2* T8473C polymorphism and meat intake in the same study (Andersen et al. 2013a, b). Thus, the biological implication of *PTGS2* on meat carcinogenesis is not readily interpretable.

*ABCB1*, *ABCC2*, and *ABCG2* encode the ATP-binding cassette (ABC) transport proteins ABCB1 (also called MDR1 and P-glycoprotein), ABCC2 and ABCG2, respectively. The ABC transporters have been found to transport a wide variety of compounds over the cell membrane, including amino acids, peptides, ions, metabolites, vitamins, fatty acid derivatives, steroids, organic anions, phospholipids, drugs, and other exogenous compounds (Quazi and Molday 2011; Coleman et al. 2013; Tarling et al. 2013). Specifically, ABCB1 has been associated with transport of endogenous pro-inflammatory signal substrates such as IL and LT (Johnstone et al. 2000; Pawlik et al. 2005a, b; Mizutani et al. 2008), whereas ABCC2 was found to transport diet- and smoke-derived carcinogens (Dietrich et al. 2001; Jedlitschky and Keppler 2002; Haimeur et al. 2004; Deeley and Cole 2006). In this review, we found that carriers of *ABCB1* C3435T homozygous wild-type and intron 3 G-rs3789243-A-variant allele were at high risk of CRC, whereas carriers of the other alleles were unaffected by meat intake. The silent *ABCB1* C3435T polymorphisms have been reported to change transport specificity and protein stability (Fung and Gottesman 2009; Fung et al. 2014), whereas the intron 3 G-rs3789243-A-variant allele has been associated with low *ABCB1* mRNA level in the intestine, thus suggesting that low level of *ABCB1* is a risk factor for CRC when eating meat (Andersen et al. 2013c). The release of IL-2, IL-4, interferon gamma, and tumour necrosis factor-alpha from activated peripheral blood mononuclear cells was found to be significantly lower among carriers of the homozygous T-variant allele of *ABCB1* C3435T compared to the carriers of the wild-type allele (Johnstone et al. 2000; Pawlik et al. 2005a, b; Mizutani et al. 2008). Thus, the results therefore suggest that genetically determined low *ABCB1* level disposes for CRC when eating meat.

Cytokines such as the pro-inflammatory IL-1B and the anti-inflammatory IL-10 are mediators of inflammation in the intestine (Coussens and Werb 2002). In this review, we found interaction between meat intake and the marker polymorphism near *IL10* rs3024505. The functional effects of rs3024505 are not known, so the interpretation of the possible biological impact in relation to meat carcinogenesis was not possible. In this review, we found no interaction between *IL1B* and meat intake, suggesting that *IL1B* is not involved in meat carcinogenesis in relation to CRC.

Transcription factors bind to DNA sequences, thereby regulating the transcription process for the targeted genes. Pregnane X receptor (PXR) and liver X receptor (LXR) are members of the nuclear receptor superfamily that regulate responses to xenobiotic exposure and lipid homeostasis, respectively (di Masi et al. 2009; McEwan 2009). Nuclear factor-kappa B (NFκB) is involved in inflammatory response, apoptosis, and cell proliferation (Seufert et al. 2013). In this review, we found that carriers of the *NFKB1* −94ins/del del-variant alleles were at high risk of CRC, whereas homozygous wild-type allele carriers had no risk by eating meat. The −94 del-variant was found to be associated with low transcription of *NFKB1* p50 in a luciferase reporter system (Karban et al. 2004). Hence, the deletion allele leads to lower levels of the p50 subunit of NFκB. This would lead to preferential depletion of the anti-inflammatory p50 dimer of NFκB, which, in turn, may lead to a relative overweight of the pro-inflammatory effects of NFκB. The results of this review therefore suggest that carriers of the *NFKB1* −94ins/del del-variant allele were at high risk of CRC due to genetically determined high inflammatory response.

Heme iron has been associated with cell proliferation in intestinal mucosa (Santarelli et al. 2008; Ferguson 2010; Alexander and Cushing 2011; Alexander et al. 2011; Erridge 2011; Zur 2012). Also, heme in red meat has been found to stimulate the production of mutagenic NOC (Joosen et al. 2009). Heme oxygenase-1 (encoded by *HMOX1*) is the rate-limiting enzyme in the degradation of heme to carbon monoxide (CO), iron, and biliverdin, thereby reducing cellular oxidative stress and inhibiting pro-inflammatory cytokines (Oates and West 2006). *HMOX1* A-413T (rs2071746) polymorphism affects heme oxygenase-1 activity (Ono et al. 2004). The assessment of interactions between meat intake and functional polymorphisms in *HMOX1* may therefore indicate whether heme or heme iron contributes to CRC risk (Tappel 2007). In this review, we found no interactions between the functional *HMOX1* A-413T (rs2071746) polymorphism and meat intake in relation to CRC. Thus, the results suggest that neither heme nor heme iron is a strong risk factor for CRC.

Meat, particularly processed meat, contains mutagens such as NOC, HCAs, and PAHs, which may increase the risk of CRC among persons with genetically determined low DNA repair capacity (Santarelli et al. 2008; Ferguson 2010; Alexander and Cushing 2011; Alexander et al. 2011; Erridge 2011; Zur 2012). Mismatch repair primarily corrects single base-pair mismatches and small insertion–deletion loops that arise during DNA replication (Berndt et al. 2007). The nucleotide excision repair (NER) pathway is the primary mechanism for repair of bulky DNA adducts and thus is an important part of the cellular defence against a large variety of structurally unrelated DNA lesions (Hansen et al. 2007). In this review, interactions between *MSH3* and *XPC* involved in DNA repair and meat in relation to CRC were suggested in prospective studies. Furthermore, interactions between the *XPC* Lys939Gln/K939Q and red meat intake were found in two independent cohorts (Table 1 and S1). Steck et al. found increased risk by high well-done red meat intake among *XPC* Lys939Gln homozygous wild-type carriers in a case–control study, whereas Sorensen in a prospective study found increased risk by red meat intake among the homozygous variant carriers compared to the homozygous wild-type carriers with low meat intake (reference group) (Hansen et al. 2007; Steck et al. 2014). Thus, the finding in the prospective cohort was not replicated in the case–control cohort. The different direction of the risk estimates between the two studies may be due to varying linkage of the *XPC* Lys939Gln polymorphism with functional polymorphisms within the same gene between the two studied populations (Aissani 2014). The functional implication of this polymorphism is not clear (Zhu et al. 2014). Thus, although the functional implications of the *XPC* polymorphism are difficult to interpret, the results suggest that meat intake leads to the formation of DNA adducts and that this mechanism is involved in meat carcinogenesis.

Some of the findings in this review point to the same underlying mechanisms. *PTGS2*, *IL10*, *ABCB1*, and *NFKB1* are all involved in the intestinal immune response, thus suggesting the involvement of the inflammatory response in meat-related carcinogenesis. Furthermore, the use of functional polymorphisms enables a biological interpretation of the interactions of *ABCB1* and *NFKB1* with meat. Interaction analyses indicated that meat intake selectively increased the risk of CRC among carriers of the *NFKB1* del-variant allele associated with high pro-inflammatory activity and among the carriers of the *ABCB1* allele associated with functional release of pro-inflammatory molecules from activated immune cells (Karban et al. 2004; Pawlik et al. 2005a, b). Therefore, these results suggest that genetically determined high inflammatory response is involved in meat colorectal carcinogenesis. Also, the suggested interaction with *MSH3* and *PXC* supports a role of DNA adducts in meat carcinogenesis. The results of this review together with recent findings thereby suggest a link between meat intake and cancer via intestinal inflammation and DNA damage (Carbonero et al. 2012; Devkota et al. 2012; Jia et al. 2012). Also, negative findings may provide important information. The present study did not support a strong role of heme, iron, and HAC cooking carcinogens in the aetiology of CRC.

The limitations of this review were derived from heterogeneity and the known large variability in meat intake and meat cooking methods between the included studies. The included case–control studies are hampered by recall bias. Recall bias may severely affect the quality of the self-reported data making the use of objective data or prospectively self-reported data desirable. Large prospective studies are needed in order to have sufficient power to assess gene–environment interactions. Also, the meat intake should be high and sufficiently distributed among the participants in the studied cohort. Seven of the eleven prospective studies were performed in the Danish “Diet, Cancer and Health” cohort, and Danes have a high meat intake compared with low-income countries. For example, *NFKB1* was associated with CRC in a Swedish cohort but not in a Chinese (Lewander et al. 2007). The results from the Danish study suggest that interaction between meat intake and *NFKB1* may be part of the reason why *NFKB1* was associated with CRC in the Swedish cohort with a high meat intake but not among Chinese who have a low intake of meat. In addition, the careful selection of functional polymorphisms or subsequent functional characterisation of polymorphisms is of most importance if biological interpretation is to be performed. Because the analyses were based on biologically funded hypothesis, we used a *P* value for the interaction of 0.05 as significance level. Traditionally, carcinogens are identified using a combination of animal studies and epidemiological studies (IARC 2014). Gene–environment interactions should be regarded a complementary approach which may prove a useful way of identifying the combinations of environmental factors and biological pathways in carcinogenesis. Future studies should aim at assessing multiple functional polymorphisms in biological pathways or networks hypothesised to affect meat carcinogenesis using large well-characterised prospective cohorts with relevant meat exposure.

All in all, we found indications from prospective studies that meat interacts with polymorphisms in *PTGS2*, *IL10*, *ABCB1*, *NFKB1*, *XPC*, and *MSH3*, but not *IL1B*, *HMOX1*, *ABCC2*, *ABCG2*, *NR1I2*, *NR1H2*, *NAT1*, *NAT2*, *MSH6*, or *MLH1* in relation to CRC (Table 1). However, none of the found interactions were replicated.

## Conclusion

The results from this systematic review suggest that genetic variation in the inflammatory response and DNA repair is involved in meat-related colorectal carcinogenesis, and no support for the involvement of heme and iron from meat or cooking mutagens was found. However, none of the found interactions had been replicated. Further studies of the biological effects by meat intake in relation to CRC are highly warranted.

## Electronic supplementary material

Below is the link to the electronic supplementary material.

Supplementary material 1 (DOCX 46 kb)

## References

[CR1] Aissani B (2014). Confounding by linkage disequilibrium. J Hum Genet.

[CR2] Alexander DD, Cushing CA (2011). Red meat and colorectal cancer: a critical summary of prospective epidemiologic studies. Obes Rev.

[CR3] Alexander DD, Weed DL, Cushing CA, Lowe KA (2011). Meta-analysis of prospective studies of red meat consumption and colorectal cancer. Eur J Cancer Prev.

[CR4] Andersen V, Vogel U (2014a) Dietary fibres and meat in relation to colorectal cancer. Norske Gastroenterologisk Forening-nytt 34–36

[CR5] Andersen V, Vogel U (2014b) Systematic review: interactions between aspirin, and other nonsteroidal anti-inflammatory drugs, and polymorphisms in relation to colorectal cancer. Aliment Pharmacol Ther 40(2):147–159. doi:10.1111/apt.12807 10.1111/apt.12807 PMC4225470 24889212

[CR6] Andersen V, Ostergaard M, Christensen J, Overvad K, Tjonneland A, Vogel U (2009). Polymorphisms in the xenobiotic transporter Multidrug Resistance 1 (MDR1) gene and interaction with meat intake in relation to risk of colorectal cancer in a Danish prospective case–cohort study. BMC Cancer.

[CR7] Andersen V, Christensen J, Overvad K, Tjonneland A, Vogel U (2010). Polymorphisms in NFkB, PXR, LXR and risk of colorectal cancer in a prospective study of Danes. BMC Cancer.

[CR8] Andersen V, Christensen J, Overvad K, Tjonneland A, Vogel U (2011). Heme oxygenase-1 polymorphism is not associated with risk of colorectal cancer: a Danish prospective study. Eur J Gastroenterol Hepatol.

[CR9] Andersen V, Nimmo E, Krarup HB, Drummond H, Christensen J, Ho GT, Ostergaard M, Ernst A, Lees C, Jacobsen BA, Satsangi J, Vogel U (2011). Cyclooxygenase-2 (COX-2) polymorphisms and risk of inflammatory bowel disease in a Scottish and Danish case–control study. Inflamm Bowel Dis.

[CR10] Andersen V, Egeberg R, Tjonneland A, Vogel U (2012). ABCC2 transporter gene polymorphisms, diet and risk of colorectal cancer: a Danish prospective cohort study. Scand J Gastroenterol.

[CR11] Andersen V, Egeberg R, Tjonneland A, Vogel U (2012). Interaction between interleukin-10 (IL-10) polymorphisms and dietary fibre in relation to risk of colorectal cancer in a Danish case–cohort study. BMC Cancer.

[CR12] Andersen V, Holst R, Kopp TI, Tjonneland A, Vogel U (2013). Interactions between diet, lifestyle and IL10, IL1B, and PTGS2/COX-2 gene polymorphisms in relation to risk of colorectal cancer in a prospective danish case–cohort study. PLoS One.

[CR13] Andersen V, Holst R, Vogel U (2013). Systematic review: diet–gene interactions and the risk of colorectal cancer. Aliment Pharmacol Ther.

[CR14] Andersen V, Vogel U, Godiksen S, Frenzel FB, Saebo M, Hamfjord J, Kure E, Vogel LK (2013). Low ABCB1 gene expression is an early event in colorectal carcinogenesis. PLoS One.

[CR15] Bacchi S, Palumbo P, Sponta A, Coppolino MF (2012). Clinical pharmacology of non-steroidal anti-inflammatory drugs: a review. Antiinflamm Antiallergy Agents Med Chem.

[CR16] Berndt SI, Platz EA, Fallin MD, Thuita LW, Hoffman SC, Helzlsouer KJ (2007). Mismatch repair polymorphisms and the risk of colorectal cancer. Int J Cancer.

[CR17] Brevik A, Joshi AD, Corral R, Onland-Moret NC, Siegmund KD, Le Marchand L, Baron JA, Martinez ME, Haile RW, Ahnen DJ, Sandler RS, Lance P, Stern MC (2010). Polymorphisms in base excision repair genes as colorectal cancer risk factors and modifiers of the effect of diets high in red meat. Cancer Epidemiol Biomark Prev.

[CR18] Brosens LA, Iacobuzio-Donahue CA, Keller JJ, Hustinx SR, Carvalho R, Morsink FH, Hylind LM, Offerhaus GJ, Giardiello FM, Goggins M (2005). Increased cyclooxygenase-2 expression in duodenal compared with colonic tissues in familial adenomatous polyposis and relationship to the -765G ->C COX-2 polymorphism. Clin Cancer Res.

[CR19] Butler LM, Millikan RC, Sinha R, Keku TO, Winkel S, Harlan B, Eaton A, Gammon MD, Sandler RS (2008). Modification by *N*-acetyltransferase 1 genotype on the association between dietary heterocyclic amines and colon cancer in a multiethnic study. Mutat Res.

[CR20] Carbonero F, Benefiel AC, Alizadeh-Ghamsari AH, Gaskins HR (2012). Microbial pathways in colonic sulfur metabolism and links with health and disease. Front Physiol.

[CR21] Chan AT, Tranah GJ, Giovannucci EL, Willett WC, Hunter DJ, Fuchs CS (2005). Prospective study of *N*-acetyltransferase-2 genotypes, meat intake, smoking and risk of colorectal cancer. Int J Cancer.

[CR22] Chan DS, Lau R, Aune D, Vieira R, Greenwood DC, Kampman E, Norat T (2011). Red and processed meat and colorectal cancer incidence: meta-analysis of prospective studies. PLoS One.

[CR23] Chen J, Stampfer MJ, Hough HL, Garcia-Closas M, Willett WC, Hennekens CH, Kelsey KT, Hunter DJ (1998). A prospective study of *N*-acetyltransferase genotype, red meat intake, and risk of colorectal cancer. Cancer Res.

[CR24] Coleman JA, Quazi F, Molday RS (2013). Mammalian P4-ATPases and ABC transporters and their role in phospholipid transport. Biochim Biophys Acta.

[CR25] Cotterchio M, Boucher BA, Manno M, Gallinger S, Okey AB, Harper PA (2008). Red meat intake, doneness, polymorphisms in genes that encode carcinogen-metabolizing enzymes, and colorectal cancer risk. Cancer Epidemiol Biomark Prev.

[CR26] Coussens LM, Werb Z (2002). Inflammation and cancer. Nature.

[CR27] Deeley RG, Cole SP (2006). Substrate recognition and transport by multidrug resistance protein 1 (ABCC1). FEBS Lett.

[CR28] Devkota S, Wang Y, Musch MW, Leone V, Fehlner-Peach H, Nadimpalli A, Antonopoulos DA, Jabri B, Chang EB (2012). Dietary-fat-induced taurocholic acid promotes pathobiont expansion and colitis in Il10-/- mice. Nature.

[CR29] di Masi A, Marinis ED, Ascenzi P, Marino M (2009). Nuclear receptors CAR and PXR: molecular, functional, and biomedical aspects. Mol Aspects Med.

[CR30] Dietrich CG, de Waart DR, Ottenhoff R, Bootsma AH, van Gennip AH, Elferink RP (2001). Mrp2-deficiency in the rat impairs biliary and intestinal excretion and influences metabolism and disposition of the food-derived carcinogen 2-amino-1-methyl-6-phenylimidazo. Carcinogenesis.

[CR31] Erridge C (2011). Accumulation of stimulants of Toll-like receptor (TLR)-2 and TLR4 in meat products stored at 5°C. J Food Sci.

[CR32] Ferguson LR (2010). Meat and cancer. Meat Sci.

[CR33] Fung KL, Gottesman MM (2009). A synonymous polymorphism in a common MDR1 (ABCB1) haplotype shapes protein function. Biochim Biophys Acta.

[CR34] Fung KL, Pan J, Ohnuma S, Lund PE, Pixley JN, Kimchi-Sarfaty C, Ambudkar SV, Gottesman MM (2014). MDR1 synonymous polymorphisms alter transporter specificity and protein stability in a stable epithelial monolayer. Cancer Res.

[CR35] Gilsing AM, Berndt SI, Ruder EH, Graubard BI, Ferrucci LM, Burdett L, Weissfeld JL, Cross AJ, Sinha R (2012). Meat-related mutagen exposure, xenobiotic metabolizing gene polymorphisms and the risk of advanced colorectal adenoma and cancer. Carcinogenesis.

[CR36] Girard H, Butler LM, Villeneuve L, Millikan RC, Sinha R, Sandler RS, Guillemette C (2008). UGT1A1 and UGT1A9 functional variants, meat intake, and colon cancer, among Caucasians and African-Americans. Mutat Res.

[CR37] IARC, h. m. i. f. (2014). “http://monographs.iarc.fr/“

[CR38] Habermann N, Ulrich CM, Lundgreen A, Makar KW, Poole EM, Caan B, Kulmacz R, Whitton J, Galbraith R, Potter JD, Slattery ML (2013). PTGS1, PTGS2, ALOX5, ALOX12, ALOX15, and FLAP SNPs: interaction with fatty acids in colon cancer and rectal cancer. Genes Nutr.

[CR39] Haimeur A, Conseil G, Deeley RG, Cole SP (2004). The MRP-related and BCRP/ABCG2 multidrug resistance proteins: biology, substrate specificity and regulation. Curr Drug Metab.

[CR40] Hamer HM, De Preter V, Windey K, Verbeke K (2012). Functional analysis of colonic bacterial metabolism: relevant to health?. Am J Physiol Gastrointest Liver Physiol.

[CR41] Hansen RD, Sorensen M, Tjonneland A, Overvad K, Wallin H, Raaschou-Nielsen O, Vogel U (2007). XPA A23G, XPC Lys939Gln, XPD Lys751Gln and XPD Asp312Asn polymorphisms, interactions with smoking, alcohol and dietary factors, and risk of colorectal cancer. Mutat Res.

[CR42] Hu X, Yuan P, Yan J, Feng F, Li X, Liu W, Yang Y (2013). Gene polymorphisms of +45T>G, −866G>A, and Ala54Thr on the risk of colorectal cancer: a matched case–control study. PLoS One.

[CR43] Hu XQ, Yuan P, Luan RS, Li XL, Liu WH, Feng F, Yan J, Yang YF (2013). Calpain-10 SNP43 and SNP19 polymorphisms and colorectal cancer: a matched case–control study. Asian Pac J Cancer Prev.

[CR44] Huxley RR, Nsary-Moghaddam A, Clifton P, Czernichow S, Parr CL, Woodward M (2009). The impact of dietary and lifestyle risk factors on risk of colorectal cancer: a quantitative overview of the epidemiological evidence. Int J Cancer.

[CR45] Jedlitschky G, Keppler D (2002). Transport of leukotriene C4 and structurally related conjugates. Vitam Horm.

[CR46] Jia W, Whitehead RN, Griffiths L, Dawson C, Bai H, Waring RH, Ramsden DB, Hunter JO, Cauchi M, Bessant C, Fowler DP, Walton C, Turner C, Cole JA (2012). Diversity and distribution of sulphate-reducing bacteria in human faeces from healthy subjects and patients with inflammatory bowel disease. FEMS Immunol Med Microbiol.

[CR47] Johnstone RW, Ruefli AA, Smyth MJ (2000). Multiple physiological functions for multidrug transporter P-glycoprotein?. Trends Biochem Sci.

[CR48] Joosen AM, Kuhnle GG, Aspinall SM, Barrow TM, Lecommandeur E, Azqueta A, Collins AR, Bingham SA (2009). Effect of processed and red meat on endogenous nitrosation and DNA damage. Carcinogenesis.

[CR49] Joshi AD, Corral R, Siegmund KD, Haile RW, Le Marchand L, Martinez ME, Ahnen DJ, Sandler RS, Lance P, Stern MC (2009). Red meat and poultry intake, polymorphisms in the nucleotide excision repair and mismatch repair pathways and colorectal cancer risk. Carcinogenesis.

[CR50] Karban AS, Okazaki T, Panhuysen CI, Gallegos T, Potter JJ, Bailey-Wilson JE, Silverberg MS, Duerr RH, Cho JH, Gregersen PK, Wu Y, Achkar JP, Dassopoulos T, Mezey E, Bayless TM, Nouvet FJ, Brant SR (2004). Functional annotation of a novel NFKB1 promoter polymorphism that increases risk for ulcerative colitis. Hum Mol Genet.

[CR51] Koh WP, Yuan JM, van den Berg D, Lee HP, Yu MC (2004). Interaction between cyclooxygenase-2 gene polymorphism and dietary n-6 polyunsaturated fatty acids on colon cancer risk: the Singapore Chinese Health Study. Br J Cancer.

[CR52] Kuriki K, Hirose K, Matsuo K, Wakai K, Ito H, Kanemitsu Y, Hirai T, Kato T, Hamajima N, Takezaki T, Suzuki T, Saito T, Tanaka R, Tajima K (2006). Meat, milk, saturated fatty acids, the Pro12Ala and C161T polymorphisms of the PPARgamma gene and colorectal cancer risk in Japanese. Cancer Sci.

[CR53] Kury S, Buecher B, Robiou-du-Pont S, Scoul C, Sebille V, Colman H, Le HC, Le NT, Bourdon J, Faroux R, Ollivry J, Lafraise B, Chupin LD, Bezieau S (2007). Combinations of cytochrome P450 gene polymorphisms enhancing the risk for sporadic colorectal cancer related to red meat consumption. Cancer Epidemiol Biomark Prev.

[CR54] Le Marchand L, Donlon T, Seifried A, Wilkens LR (2002). Red meat intake, CYP2E1 genetic polymorphisms, and colorectal cancer risk. Cancer Epidemiol Biomark Prev.

[CR55] Lewander A, Butchi AK, Gao J, He LJ, Lindblom A, Arbman G, Carstensen J, Zhang ZY, Sun XF (2007). Polymorphism in the promoter region of the NFKB1 gene increases the risk of sporadic colorectal cancer in Swedish but not in Chinese populations. Scand J Gastroenterol.

[CR56] Little J, Sharp L, Masson LF, Brockton NT, Cotton SC, Haites NE, Cassidy J (2006). Colorectal cancer and genetic polymorphisms of CYP1A1, GSTM1 and GSTT1: a case–control study in the Grampian region of Scotland. Int J Cancer.

[CR57] McEwan IJ (2009). Nuclear receptors: one big family. Methods Mol Biol.

[CR58] Mizutani T, Masuda M, Nakai E, Furumiya K, Togawa H, Nakamura Y, Kawai Y, Nakahira K, Shinkai S, Takahashi K (2008). Genuine functions of P-glycoprotein (ABCB1). Curr Drug Metab.

[CR59] Moher D, Liberati A, Tetzlaff J, Altman DG (2009). Preferred reporting items for systematic reviews and meta-analyses: the PRISMA statement. PLoS Med.

[CR60] Moore AE, Young LE, Dixon DA (2012). A common single-nucleotide polymorphism in cyclooxygenase-2 disrupts microRNA-mediated regulation. Oncogene.

[CR61] Morita M, Le Marchand L, Kono S, Yin G, Toyomura K, Nagano J, Mizoue T, Mibu R, Tanaka M, Kakeji Y, Maehara Y, Okamura T, Ikejiri K, Futami K, Maekawa T, Yasunami Y, Takenaka K, Ichimiya H, Imaizumi N (2009). Genetic polymorphisms of CYP2E1 and risk of colorectal cancer: the Fukuoka Colorectal Cancer Study. Cancer Epidemiol Biomark Prev.

[CR62] Nothlings U, Yamamoto JF, Wilkens LR, Murphy SP, Park SY, Henderson BE, Kolonel LN, Le Marchand L (2009). Meat and heterocyclic amine intake, smoking, NAT1 and NAT2 polymorphisms, and colorectal cancer risk in the multiethnic cohort study. Cancer Epidemiol Biomark Prev.

[CR63] Oates PS, West AR (2006). Heme in intestinal epithelial cell turnover, differentiation, detoxification, inflammation, carcinogenesis, absorption and motility. World J Gastroenterol.

[CR64] Ollberding NJ, Wilkens LR, Henderson BE, Kolonel LN, Le Marchand L (2012). Meat consumption, heterocyclic amines and colorectal cancer risk: the Multiethnic Cohort Study. Int J Cancer.

[CR65] Ono K, Goto Y, Takagi S, Baba S, Tago N, Nonogi H, Iwai N (2004). A promoter variant of the heme oxygenase-1 gene may reduce the incidence of ischemic heart disease in Japanese. Atherosclerosis.

[CR66] Papafili A, Hill MR, Brull DJ, McAnulty RJ, Marshall RP, Humphries SE, Laurent GJ (2002). Common promoter variant in cyclooxygenase-2 represses gene expression: evidence of role in acute-phase inflammatory response. Arterioscler Thromb Vasc Biol.

[CR67] Pawlik A, Baskiewicz-Masiuk M, Machalinski B, Kurzawski M, Gawronska-Szklarz B (2005). Involvement of C3435T and G2677T multidrug resistance gene polymorphisms in release of cytokines from peripheral blood mononuclear cells treated with methotrexate and dexamethasone. Eur J Pharmacol.

[CR68] Pawlik A, Baskiewicz-Masiuk M, Machalinski B, Safranow K, Gawronska-Szklarz B (2005). Involvement of P-glycoprotein in the release of cytokines from peripheral blood mononuclear cells treated with methotrexate and dexamethasone. J Pharm Pharmacol.

[CR69] Quazi F, Molday RS (2011). Lipid transport by mammalian ABC proteins. Essays Biochem.

[CR70] Santarelli RL, Pierre F, Corpet DE (2008). Processed meat and colorectal cancer: a review of epidemiologic and experimental evidence. Nutr Cancer.

[CR71] Seufert BL, Poole EM, Whitton J, Xiao L, Makar KW, Campbell PT, Kulmacz RJ, Baron JA, Newcomb PA, Slattery ML, Potter JD, Ulrich CM (2013). IkappaBKbeta and NFkappaB1, NSAID use and risk of colorectal cancer in the Colon Cancer Family Registry. Carcinogenesis.

[CR72] Slattery ML, Samowitz W, Ballard L, Schaffer D, Leppert M, Potter JD (2001). A molecular variant of the APC gene at codon 1822: its association with diet, lifestyle, and risk of colon cancer. Cancer Res.

[CR73] Sorensen M, Autrup H, Olsen A, Tjonneland A, Overvad K, Raaschou-Nielsen O (2008). Prospective study of NAT1 and NAT2 polymorphisms, tobacco smoking and meat consumption and risk of colorectal cancer. Cancer Lett.

[CR74] Steck SE, Butler LM, Keku T, Antwi S, Galanko J, Sandler RS, Hu JJ (2014). Nucleotide excision repair gene polymorphisms, meat intake and colon cancer risk. Mutat Res Fundam Mol Mech Mutagen.

[CR75] Tappel A (2007). Heme of consumed red meat can act as a catalyst of oxidative damage and could initiate colon, breast and prostate cancers, heart disease and other diseases. Med Hypotheses.

[CR76] Tarling EJ, de Aguiar Vallim TQ, Edwards PA (2013). Role of ABC transporters in lipid transport and human disease. Trends Endocrinol Metab.

[CR77] Theodoratou E, Campbell H, Tenesa A, McNeill G, Cetnarskyj R, Barnetson RA, Porteous ME, Dunlop MG, Farrington SM (2008). Modification of the associations between lifestyle, dietary factors and colorectal cancer risk by APC variants. Carcinogenesis.

[CR78] Tiemersma EW, Kampman E, Bueno de Mesquita HB, Bunschoten A, van Schothorst EM, Kok FJ, Kromhout D (2002). Meat consumption, cigarette smoking, and genetic susceptibility in the etiology of colorectal cancer: results from a Dutch prospective study. Cancer Causes Control.

[CR79] Turner F, Smith G, Sachse C, Lightfoot T, Garner RC, Wolf CR, Forman D, Bishop DT, Barrett JH (2004). Vegetable, fruit and meat consumption and potential risk modifying genes in relation to colorectal cancer. Int J Cancer.

[CR80] Vogel U, Christensen J, Dybdahl M, Friis S, Hansen RD, Wallin H, Nexo BA, Raaschou-Nielsen O, Andersen PS, Overvad K, Tjonneland A (2007). Prospective study of interaction between alcohol, NSAID use and polymorphisms in genes involved in the inflammatory response in relation to risk of colorectal cancer. Mutat Res.

[CR81] Wang D, DuBois RN (2010). Eicosanoids and cancer. Nat Rev Cancer.

[CR82] Wang D, DuBois RN (2010). The role of COX-2 in intestinal inflammation and colorectal cancer. Oncogene.

[CR83] Wang D, DuBois RN (2013). Urinary PGE-M: a promising cancer biomarker. Cancer Prev Res (Phila).

[CR84] Wang J, Joshi AD, Corral R, Siegmund KD, Marchand LL, Martinez ME, Haile RW, Ahnen DJ, Sandler RS, Lance P, Stern MC (2012). Carcinogen metabolism genes, red meat and poultry intake, and colorectal cancer risk. Int J Cancer.

[CR85] WCRF, W. C. R. F. (2014, 1/22/2014). World Cancer Research Fund International http://www.wcrf.org/World Cancer Research Fund International

[CR86] Windey K, De Preter V, Verbeke K (2012). Relevance of protein fermentation to gut health. Mol Nutr Food Res.

[CR87] Yeh CC, Hsieh LL, Tang R, Chang-Chieh CR, Sung FC (2005). MS-920: DNA repair gene polymorphisms, diet and colorectal cancer risk in Taiwan. Cancer Lett.

[CR88] Yeh CC, Sung FC, Tang R, Chang-Chieh CR, Hsieh LL (2009). Polymorphisms of cytochrome P450 1A2 and *N*-acetyltransferase genes, meat consumption, and risk of colorectal cancer. Dis Colon Rectum.

[CR89] Zhang X, Miao X, Tan W, Ning B, Liu Z, Hong Y, Song W, Guo Y, Zhang X, Shen Y, Qiang B, Kadlubar FF, Lin D (2005). Identification of functional genetic variants in cyclooxygenase-2 and their association with risk of esophageal cancer. Gastroenterology.

[CR90] Zhu ML, Hua RX, Zheng L (2014). Associations between polymorphisms of the XPC gene and lung cancer susceptibility: a meta-analysis. Tumour Biol.

[CR91] Zur H (2012). Red meat consumption and cancer: reasons to suspect involvement of bovine infectious factors in colorectal cancer. Int J Cancer.

