# Supplementary material for: Interactions between meat intake and genetic variation in relation to colorectal cancer
Source: Genes Nutr. 2014 Dec 10;10(1):448. doi: 10.1007/s12263-014-0448-9 (PMC4261072; doi:10.1007/s12263-014-0448-9)
Supplement: Supplementary file 1 — Supplementary material 1 (DOCX 46 kb) [file 12263_2014_448_MOESM1_ESM.docx]

Interactions between Meat Intake and Genetic Variation in relation to Colorectal Cancer,

Genes & Nutrition

Vibeke Andersen^1,2,3^ and Ulla Vogel^4^

^1^Organ Center, Hospital of Southern Jutland, Aabenraa, Denmark, ^2^Institute of Regional Health Research, University of Southern Denmark, Odense, Denmark, ^3^Medical Department, Regional Hospital Viborg, Viborg, Denmark, ^4^National Research Centre for the Working Environment, Copenhagen, Denmark

Corresponding Author: Vibeke Andersen, Organ Center, Kristen Philipsens Vej 15, DK-6200 Åbenrå, Denmark, Phone: +45 2115 7790, Fax: +45 8883 4488, Email: vandersen@health.sdu.dk

**TABLE S1.** Retrospective studies on interactions between meat intake and polymorphisms in relation to colorectal cancer.

| ***Gene*** | **SNP/nucleotide** | **Rs-number^1^** | **Design** | **N_cases_** | **N_contr_** | **P_interaction_** | **comm** | **Meat type** | **Author** | **Year** | **Ref** |
| --- | --- | --- | --- | --- | --- | --- | --- | --- | --- | --- | --- |
| ***Carcinogen metabolism*** | | | | | | | | | | | |
| ***CYP1A2*** | G-3860A |  | case-control | 727 | 736 | 0.61 |  | White meat | Yeh | 2009 | [1] |
| ***NAT1*** | Slow/fast |  | case-control | 727 | 736 | 0.07 |  | White meat | Yeh | 2009 | [1] |
| ***NAT2*** | Slow/fast |  | case-control | 727 | 736 | 0.36 |  | White meat | Yeh | 2009 | [1] |
| ***CYP1A2*** | G-3860A |  | case-control | 727 | 736 | 0.22 |  | Red meat | Yeh | 2009 | [1] |
| ***NAT1*** | Slow/fast |  | case-control | 727 | 736 | 0.24 |  | Red meat | Yeh | 2009 | [1] |
| ***NAT2*** | Slow/fast |  | case-control | 727 | 736 | 0.74 |  | Red meat | Yeh | 2009 | [1] |
| ***CYP2E1*** | Rsa1 |  | case-control | 685 | 778 | 0.81 |  | Red meat | Morita | 2009 | [2] |
|  | 96-bp ins |  | case-control | 685 | 778 | 0.08 |  | Red meat | Morita | 2009 | [2] |
| ***NAT2*** |  |  | case-only | 577 |  | *0.01* |  | Red meat | Wang | 2012 | [3] |
| ***CYP1A2*** | C163A | Rs762551 | case-control | 842 | 1251 | ns |  | Red meat | Cotterchio | 2008 | [4] |
| ***CYP2E1*** | G1293C | Rs3813867 | case-control | 842 | 1251 | ns |  | Red meat | Cotterchio | 2008 | [4] |
|  | T7632A | Rs6413432 | case-control | 842 | 1251 | ns |  | Red meat | Cotterchio | 2008 | [4] |
| ***CYP2C9*** | C430T | Rs1799853 | case-control | 842 | 1251 | ns |  | Red meat | Cotterchio | 2008 | [4] |
|  | A1075C | Rs1057910 | case-control | 842 | 1251 | ns |  | Red meat | Cotterchio | 2008 | [4] |
| ***CYP1A1*** | A2455G | Rs1048943 | case-control | 842 | 1251 | ns |  | Red meat | Cotterchio | 2008 | [4] |
|  | T3801C | Rs4646903 | case-control | 842 | 1251 | ns |  | Red meat | Cotterchio | 2008 | [4] |
| ***CYP1B1*** | C142G | Rs10012 | case-control | 842 | 1251 | ns |  | Red meat | Cotterchio | 2008 | [4] |
|  | C4326G | Rs1056836 | case-control | 842 | 1251 | ns |  | Red meat | Cotterchio | 2008 | [4] |
|  | A4390G | Rs1800440 | case-control | 842 | 1251 | ns |  | Red meat | Cotterchio | 2008 | [4] |
| ***GSTM*** | GSTM3delAGG | Rs1799735 | case-control | 842 | 1251 | ns |  | Red meat | Cotterchio | 2008 | [4] |
| ***UGT1A7*** | W208R | Rs11692021 | case-control | 842 | 1251 | ns |  | Red meat | Cotterchio | 2008 | [4] |
|  | N129K | Rs17868323 | case-control | 842 | 1251 | ns |  | Red meat | Cotterchio | 2008 | [4] |
| ***UGT1A1*28*** | A(TA)6TAA>A(TA)7TAA |  | case-control | 842 | 1251 | ns |  | Red meat | Cotterchio | 2008 | [4] |
| ***mEH*** | T17673C | Rs1051740 | case-control | 842 | 1251 | ns |  | Red meat | Cotterchio | 2008 | [4] |
| ***SULT1A1*** | G638A | Rs928261 | case-control | 842 | 1251 | ns |  | Red meat | Cotterchio | 2008 | [4] |
| ***NAT1*** | G459A | Rs4986990 | case-control | 842 | 1251 | ns |  | Red meat | Cotterchio | 2008 | [4] |
|  | T1088A | Rs8190861 | case-control | 842 | 1251 | ns |  | Red meat | Cotterchio | 2008 | [4] |
| ***NAT2*** | T341C | Rs1801280 | case-control | 842 | 1251 | ns |  | Red meat | Cotterchio | 2008 | [4] |
|  | G590A | Rs1799930 | case-control | 842 | 1251 | ns |  | Red meat | Cotterchio | 2008 | [4] |
|  | G857A | Rs1799931 | case-control | 842 | 1251 | ns |  | Red meat | Cotterchio | 2008 | [4] |
| ***ARH*** | G1661A | Rs2066853 | case-control | 842 | 1251 | ns |  | Red meat | Cotterchio | 2008 | [4] |
| ***UGT1A1*** | -53 |  | case-control | 537 | 866 | 0.37 | 3 | Red meat | Girard | 2008 | [5] |
| ***UGT1A9*** | -3156 |  | case-control | 537 | 866 | 0.65 | 3 | Red meat | Girard | 2008 | [5] |
| ***CYP1A2*** | A163C | Rs762551 | Case-control | 1023 | 1121 | ns |  | Red meat | Küry | 2007 | [6] |
|  | T1548C | Rs2470890 | Case-control | 1023 | 1121 | ns |  | Red meat | Küry | 2007 | [6] |
| ***CYP2E1*** | G-1293C | Rs3813867 | Case-control | 1023 | 1121 | ns |  | Red meat | Küry | 2007 | [6] |
|  | C-1053T | Rs2031920 | Case-control | 1023 | 1121 | ns |  | Red meat | Küry | 2007 | [6] |
| ***CYP1B1*** | C1294G | Rs1056836 | Case-control | 1023 | 1121 | ns |  | Red meat | Küry | 2007 | [6] |
| ***CYP2C9*** | C430T | Rs1799853 | Case-control | 1023 | 1121 | ns |  | Red meat | Küry | 2007 | [6] |
| ***CYP1A1*** | m1 |  | case-control | 264 | 408 | *0.049* |  | Meat | Little | 2006 | [7] |
| ***GSTM1*** | m2 |  | case-control | 264 | 408 | *0.032* |  | Meat | Little | 2006 | [7] |
| ***GSTT1*** | m4 |  | case-control | 264 | 408 | *0.023* |  | Meat | Little | 2006 | [7] |
| ***GSTM1*** | na |  | case-control | 727 | 736 | ns |  | meat | Yeh | 2005 | [8] |
| ***GSTP1*** | Ile105Val | Rs1695^1^ | case-control | 727 | 736 | ns |  | meat | Yeh | 2005 | [8] |
| ***GSTT1*** | na |  | case-control | 727 | 736 | ns |  | meat | Yeh | 2005 | [8] |
| ***GSTP*** | Ile105Val | Rs1695^1^ | case-control | 484 | 738 | *0.02* |  | red meat | Turner | 2004 | [9] |
| ***GSTP*** | Ala114Val | Rs1138272 | case-control | 484 | 738 | 0.27 |  | red meat | Turner | 2004 | [9] |
| ***EPHX1*** | His139Arg | rs2234922^1^ | case-control | 484 | 738 | 0.62 |  | red meat | Turner | 2004 | [9] |
| ***CYP1A1*** | E7 |  | case-control | 484 | 738 | 0.88 |  | red meat | Turner | 2004 | [9] |
| ***CYP1A1*** | Msp |  | case-control | 484 | 738 | 0.93 |  | red meat | Turner | 2004 | [9] |
| ***NQO1*** | Pro187Ser |  | case-control | 484 | 738 | 0.32 |  | red meat | Turner | 2004 | [9] |
| ***NQO1*** | Arg 139Trp | Rs1131341^1^ | case-control | 484 | 738 | 0.14 |  | red meat | Turner | 2004 | [9] |
| ***CYP2E1*** | RsaI |  | case-control | 174 | 315 | 0.18 | 4 | red meat | Marchand | 2002 | [10] |
| ***CYP2E1*** | 96-bp ins |  | case-control | 174 | 315 | 0.28 | 4 | red meat | Marchand | 2002 | [10] |
| ***CYP2E1*** | RsaI |  | case-control | 65 | 315 | 0.64 | 5 | red meat | Marchand | 2002 | [10] |
| ***CYP2E1*** | 96-bp ins |  | case-control | 64 | 315 | 0.30 | 5 | red meat | Marchand | 2002 | [10] |
| ***CYP1A1*** | *1/*2 |  | Case-control | 1346 | 1544 | ns | 7 | Red meat | Murtaugh | 2005 | [11] |
| ***CYP1A1*** | *1/*2 |  | Case-control | 952 | 1544 | ns | 7 | Red meat | Murtaugh | 2005 | [11] |
| ***Hormonal effects*** | | | | | | | | | | | |
| ***CAPN10*** | Snp 43 |  | case-control | 400 | 400 |  | 2 | Red meat | Hu | 2013 | [12] |
|  | Snp 19 |  | case-control | 400 | 400 |  | 2 | Red meat | Hu | 2013 | [12] |
| ***ADIPOQ*** | T45G | Rs2241766 | case-control | 400 | 400 |  | 2 | Red meat | Hu | 2013 | [13] |
| ***UCP2*** | G866A | Rs659366 | case-control | 400 | 400 |  | 2 | Red meat | Hu | 2013 | [13] |
| ***FABP2*** | Ala54Thr | Rs1799883 | case-control | 400 | 400 |  | 2 | Red meat | Hu | 2013 | [13] |
| ***DNA repair*** | | | | | | | | | | | |
| ***APEX1*** | Gln51His | Rs1048945^1^ | case-only | 577 |  | ns |  | Red meat | Brevik | 2010 | [14] |
|  | Asp148Glu | Rs1130409^9^ | case-only | 577 |  | ns |  | Red meat | Brevik | 2010 | [14] |
| ***OGG1*** | Ser236Cys |  | case-only | 577 |  | ns |  | Red meat | Brevik | 2010 | [14] |
| ***PARP*** | Val742Ala |  | case-only | 577 |  | *0.026* |  | Red meat | Brevik | 2010 | [14] |
| ***XRCC1*** | Arg194Trp | Rs1799782^1^ | case-only | 577 |  | ns |  | Red meat | Brevik | 2010 | [14] |
|  | Arg280His | Rs25489^1^ | case-only | 577 |  | ns |  | Red meat | Brevik | 2010 | [14] |
|  | Arg399Gln | Rs25487^1^ | case-only | 577 |  | ns |  | Red meat | Brevik | 2010 | [14] |
| ***MLH1*** | Ile219Val | Rs1799977 | case-control | 577 | 307 | ns | 6 | Red meat | Joshi | 2008 | [15] |
| ***MSH2*** | Gly322Asp | Rs4987188 | case-control | 577 | 307 | ns | 6 | Red meat | Joshi | 2008 | [15] |
| ***ERCC1*** | 3’UTR | Rs3212986 | case-control | 577 | 307 | ns | 6 | Red meat | Joshi | 2008 | [15] |
| ***XPD*** | Asp312Asn | Rs1799793 | case-control | 577 | 307 | ns | 6 | Red meat | Joshi | 2008 | [15] |
|  | Lys751Gln | Rs13181 | case-control | 577 | 307 | ns | 6 | Red meat | Joshi | 2008 | [15] |
| ***XPC*** | Intron 11 | Rs2279017 | case-control | 577 | 307 | ns | 6 | Red meat | Joshi | 2008 | [15] |
| ***XPA*** | 5’UTR | Rs1800975 | case-control | 577 | 307 | ns | 6 | Red meat | Joshi | 2008 | [15] |
| ***XPF*** | Arg415Gln | Rs1800067 | case-control | 577 | 307 | ns | 6 | Red meat | Joshi | 2008 | [15] |
| ***XPG*** | Asp1104His | Rs17655 | case-control | 577 | 307 | ns | 6 | Red meat | Joshi | 2008 | [15] |
| ***XRCC1*** | Arg399Gln | Rs25487^1^ | case-control | 727 | 736 | 0.43 |  | meat | Yeh | 2005 | [16] |
| ***XRCC3*** | Thr241Met | Rs79874791^1^ | case-control | 727 | 736 | *0.02* |  | meat | Yeh | 2005 | [16] |
| ***XPD*** | Lys751Gln | Rs13181^1^ | case-control | 727 | 736 | 0.47 |  | meat | Yeh | 2005 | [16] |
| ***XPD/ERCC2*** | D312N | Rs1799793^1^ | Case-control | 413 | 536 | ns |  | Well-done red meat | Steck | 2014 | [17] |
| ***XPD/ERCC2*** | K751Q | Rs13181^1^ | Case-control | 413 | 536 | ns |  | Well-done red meat | Steck | 2014 | [17] |
| ***XPF/ERCC4*** | R415Q | Rs1800067^1^ | Case-control | 413 | 536 | ns |  | Well-done red meat | Steck | 2014 | [17] |
| ***XPG/ERCC5*** | D1104H | Rs17655^1^ | Case-control | 413 | 536 | ns |  | Well-done red meat | Steck | 2014 | [17] |
| ***XPC*** | A499V | Rs2228000^1^ | Case-control | 413 | 536 | ns |  | Well-done red meat | Steck | 2014 | [17] |
| ***XPC*** | K939Q | Rs2228001^1^ | Case-control | 413 | 536 | 0.05 | 8 | Well-done red meat | Steck | 2014 | [17] |
| ***RAD23B*** | A249V |  | Case-control | 413 | 536 | ns |  | Well-done red meat | Steck | 2014 | [17] |
| ***Tumor suppression*** | | | | | | | | | | | |
| ***APC*** | Asp1822Val |  | case-only | 1656 |  | 0.11 |  | meat | Theodoratou | 2008 | [18] |
| ***APC*** | Asp1822Val |  | case-only | 1656 |  | *0.002* |  | red meat | Theodoratou | 2008 | [18] |
| ***APC*** | Glu1319Gln |  | case-only | 1656 |  | 0.84 |  | meat | Theodoratou | 2008 | [18] |
| ***APC*** | Glu1319Gln |  | case-only | 1656 |  | 0.24 |  | red meat | Theodoratou | 2008 | [18] |
| ***APC*** | B1822V |  | Case-control | 1585 | 1945 | ns |  | Red meat | Slattery | 2001 | [19] |

^1^ rs number was not provided by the authors. Rs number has been identified as described in the method section and provided if the rs number could be unambiguously identified.

^2^ Interaction was analysed by GMDR

^3^ Analyses of <28.5 g/d versus >=28.5 g/d

^4^ Analyses of red meat (beef, pork, veal, lamb) (<=47.4 g/d versus >47.4 g/d) in relation to colon cancer

^5^ Red meat (beef, pork, veal, lamb) was analysed in relation to rectum cancer

^6^ Both case-only and case-sibling design were used

^7^ Number of servings, data separate for men and women

^8^ Dominant model P_int_=0.05, recessive model P_int_=0.15

Reference List

1. Yeh CC, Sung FC, Tang R, Chang-Chieh CR, Hsieh LL: **Polymorphisms of cytochrome P450 1A2 and N-acetyltransferase genes, meat consumption, and risk of colorectal cancer.** *DisColon Rectum* 2009, **52:**104-111.

2. Morita M, Le Marchand L, Kono S, Yin G, Toyomura K, Nagano J, Mizoue T, Mibu R, Tanaka M, Kakeji Y, et al: **Genetic polymorphisms of CYP2E1 and risk of colorectal cancer: the Fukuoka Colorectal Cancer Study.** *Cancer Epidemiol Biomarkers Prev* 2009, **18:**235-241.

3. Wang J, Joshi AD, Corral R, Siegmund KD, Marchand LL, Martinez ME, Haile RW, Ahnen DJ, Sandler RS, Lance P, Stern MC: **Carcinogen metabolism genes, red meat and poultry intake, and colorectal cancer risk.** *IntJCancer* 2012, **130:**1898-1907.

4. Cotterchio M, Boucher BA, Manno M, Gallinger S, Okey AB, Harper PA: **Red meat intake, doneness, polymorphisms in genes that encode carcinogen-metabolizing enzymes, and colorectal cancer risk.** *Cancer EpidemiolBiomarkers Prev* 2008, **17:**3098-3107.

5. Girard H, Butler LM, Villeneuve L, Millikan RC, Sinha R, Sandler RS, Guillemette C: **UGT1A1 and UGT1A9 functional variants, meat intake, and colon cancer, among Caucasians and African-Americans.** *MutatRes* 2008, **644:**56-63.

6. Kury S, Buecher B, Robiou-du-Pont S, Scoul C, Sebille V, Colman H, Le HC, Le NT, Bourdon J, Faroux R, et al: **Combinations of cytochrome P450 gene polymorphisms enhancing the risk for sporadic colorectal cancer related to red meat consumption.** *Cancer EpidemiolBiomarkers Prev* 2007, **16:**1460-1467.

7. Little J, Sharp L, Masson LF, Brockton NT, Cotton SC, Haites NE, Cassidy J: **Colorectal cancer and genetic polymorphisms of CYP1A1, GSTM1 and GSTT1: a case-control study in the Grampian region of Scotland.** *IntJCancer* 2006, **119:**2155-2164.

8. Yeh CC, Hsieh LL, Tang R, Chang-Chieh CR, Sung FC: **Vegetable/fruit, smoking, glutathione S-transferase polymorphisms and risk for colorectal cancer in Taiwan.** *World J Gastroenterol* 2005, **11:**1473-1480.

9. Turner F, Smith G, Sachse C, Lightfoot T, Garner RC, Wolf CR, Forman D, Bishop DT, Barrett JH: **Vegetable, fruit and meat consumption and potential risk modifying genes in relation to colorectal cancer.** *IntJCancer* 2004, **112:**259-264.

10. Le Marchand L, Donlon T, Seifried A, Wilkens LR: **Red meat intake, CYP2E1 genetic polymorphisms, and colorectal cancer risk.** *Cancer Epidemiol Biomarkers Prev* 2002, **11:**1019-1024.

11. Murtaugh MA, Sweeney C, Ma KN, Caan BJ, Slattery ML: **The CYP1A1 genotype may alter the association of meat consumption patterns and preparation with the risk of colorectal cancer in men and women.** *J Nutr* 2005, **135:**179-186.

12. Hu XQ, Yuan P, Luan RS, Li XL, Liu WH, Feng F, Yan J, Yang YF: **Calpain-10 SNP43 and SNP19 Polymorphisms and Colorectal Cancer: a Matched Case-control Study.** *Asian PacJCancer Prev* 2013, **14:**6673-6680.

13. Hu X, Yuan P, Yan J, Feng F, Li X, Liu W, Yang Y: **Gene Polymorphisms of +45T>G, -866G>A, and Ala54Thr on the Risk of Colorectal Cancer: A Matched Case-Control Study.** *PLoSONE* 2013, **8:**e67275.

14. Brevik A, Joshi AD, Corral R, Onland-Moret NC, Siegmund KD, Le Marchand L, Baron JA, Martinez ME, Haile RW, Ahnen DJ, et al: **Polymorphisms in base excision repair genes as colorectal cancer risk factors and modifiers of the effect of diets high in red meat.** *Cancer Epidemiol Biomarkers Prev* 2010, **19:**3167-3173.

15. Joshi AD, Corral R, Siegmund KD, Haile RW, Le Marchand L, Martinez ME, Ahnen DJ, Sandler RS, Lance P, Stern MC: **Red meat and poultry intake, polymorphisms in the nucleotide excision repair and mismatch repair pathways and colorectal cancer risk.** *Carcinogenesis* 2009, **30:**472-479.

16. Yeh CC, Hsieh LL, Tang R, Chang-Chieh CR, Sung FC: **MS-920: DNA repair gene polymorphisms, diet and colorectal cancer risk in Taiwan.** *Cancer Lett* 2005, **224:**279-288.

17. Steck SE, Butler LM, Keku T, Antwi S, Galanko J, Sandler RS, Hu JJ: **Nucleotide excision repair gene polymorphisms, meat intake and colon cancer risk.** *Mutat Res Fundam Mol Mech Mutagen* 2014, **762:**24-31.

18. Theodoratou E, Campbell H, Tenesa A, McNeill G, Cetnarskyj R, Barnetson RA, Porteous ME, Dunlop MG, Farrington SM: **Modification of the associations between lifestyle, dietary factors and colorectal cancer risk by APC variants.** *Carcinogenesis* 2008, **29:**1774-1780.

19. Slattery ML, Samowitz W, Ballard L, Schaffer D, Leppert M, Potter JD: **A molecular variant of the APC gene at codon 1822: its association with diet, lifestyle, and risk of colon cancer.** *Cancer Res* 2001, **61:**1000-1004.
